# Efficacy of Single-Task Versus Dual-Task Training on Motor Function in Stroke Survivors

**DOI:** 10.7759/cureus.105600

**Published:** 2026-03-21

**Authors:** Moayanger P Ao, Shipra Chaudhary, Chethan Channaveera, Rajendra Sharma

**Affiliations:** 1 Physical Medicine and Rehabilitation, Atal Bihari Vajpayee Institute of Medical Sciences and Dr. Ram Manohar Lohia Hospital, New Delhi, IND; 2 Physical Medicine and Rehabilitation, Employees' State Insurance Corporation (ESIC) Medical College and Hospital, Faridabad, Faridabad, IND

**Keywords:** cognitive, dual task, motor, single task, stroke

## Abstract

Background: Stroke is a leading cause of long-term disability globally, frequently resulting in substantial motor and cognitive impairments. Rehabilitation approaches that integrate cognitive and motor training, such as dual-task training, have the potential to improve recovery outcomes by minimizing dual-task interference.

Objective: To evaluate the efficacy of dual-task training, which combines cognitive and motor tasks performed simultaneously, compared with single-task motor training in stroke survivors.

Methods: This prospective, randomized, comparative study enrolled 60 stroke survivors, who were randomly assigned to either the dual-task training group (n = 30) or the single-task training group (n = 30). Both groups underwent respective training protocols three times a week for four weeks. Primary outcome measures included the Timed Up and Go Test (TUG), the 10-Meter Walk Test (10MWT), and the Figure of 8 Walk Test (F8WT), assessed at baseline, four weeks, and eight weeks.

Results: The dual-task group demonstrated statistically significant improvements in all outcome measures compared to the single-task group. A significant difference was observed in the TUG at four weeks (p = 0.035). Similarly, the dual-task group showed superior gains in gait performance as measured by the 10MWT at eight weeks (p = 0.021) and in walking coordination and balance assessed by the F8WT at both four and eight weeks (p = 0.042 and p = 0.039). These findings indicate that dual-task training provides greater functional improvement than single-task training in stroke survivors.

Conclusion: Incorporating cognitive elements into motor rehabilitation through dual-task training significantly improves motor function outcomes in stroke survivors compared to single-task training alone. Rehabilitation protocols for stroke survivors should consider integrating dual-task protocols to optimize recovery and functional independence in this population.

## Introduction

The World Health Organization defined stroke as "rapidly developed clinical signs of focal (or global) disturbance of cerebral function, lasting more than 24 hours or leading to death, with no apparent cause other than of vascular origin" [1]. Stroke is a major global health problem and remains one of the leading causes of mortality and long-term disability worldwide. According to the Global Burden of Disease Study, stroke is the second leading cause of death and a major contributor to disability-adjusted life years (DALYs), affecting millions of individuals each year across various age groups [2]. Stroke frequently leads to significant motor impairments, including deficits in gait and postural control, which substantially hinder the performance of basic activities of daily living (ADLs) [3]. The complexity of these impairments requires rehabilitation strategies that focus not only on treating motor deficits but also accommodating the impaired executive cognitive function and multitasking demands during ADLs [4].

Independent ambulation requires a high level of cognitive-motor integration, including attention shifting, motor planning, obstacle negotiation, and multitasking. Post-stroke, this integration is compromised, manifesting as dual-task interference (DTI). Research over the past decade has increasingly focused on the efficacy of dual-task training that simultaneously addresses cognitive and motor functions by having patients perform two tasks concurrently (e.g., walking while counting backwards). This method has been shown to reduce DTI, which occurs when the performance of one or both tasks deteriorates relative to performing them individually [5-8]. Dual-task training aims to improve the automatization of tasks and increase the capacity to process multiple streams of information, thereby aiding the recovery of motor functions such as walking and the overall dynamic balance of stroke survivors [9,10].

While the efficacy of dual-task training is well recognized, single-task training, focusing on the repeated practice of a single task at a time, also offers substantial benefits. This method is believed to promote precise motor skill acquisition by reinforcing and correcting motor pathways [11]. Nonetheless, comparative investigations into the relative effectiveness of dual-task versus single-task training remain limited, particularly across diverse populations and various phases of stroke rehabilitation.

Most existing studies on dual-task and single-task training have been conducted in East Asian and Western populations, and very little data from Indian rehabilitation settings, where environmental constraints (e.g., limited home space) may influence functional training adaptations. By contrasting the effects of single-task and dual-task training on motor function improvement among stroke patients in an Indian context, this study seeks to close the gap and offer insights that may lead to more specialized and effective training protocols.

The objective of this study was to evaluate the efficacy of dual-task training, which combines simultaneous cognitive and motor tasks, compared with single-task motor training in improving functional mobility and motor outcomes in stroke survivors.

## Materials and methods

This prospective, randomized comparative study was conducted at the Department of Physical Medicine and Rehabilitation of a tertiary teaching hospital in North India. The study was approved by the Institutional Review Board and registered with the Clinical Trial Registry of India (CTRI/2022/12/048076). The study was conducted between August 1, 2022, and February 29, 2024. Informed consent was obtained from all individual participants involved in the study. All procedures performed were in accordance with the ethical standards of the institutional and/or national research committee and with the 1964 Helsinki Declaration and its later amendments, or with comparable ethical standards.

Study population

Sixty stroke survivors who were medically stable and had experienced a stroke (ischemic or hemorrhagic) at least six weeks prior were enrolled.

Participants were 18 years or older, able to walk at least 10 meters independently, and capable of understanding and following instructions. Cognitive ability was assessed clinically through orientation, comprehension, and the ability to follow two-step commands. Only participants able to provide informed consent and participate in training were included. Individuals with neurological conditions other than stroke, significant co-morbidities such as fractures or amputations, or severe auditory or visual impairments were excluded.

Sample size calculation

In the study by Kim et. al. [9], the mean and standard deviation (SD) for dual-task and single-task training groups based on Stroop test performance were 52.1 ± 16.62 and 34.2 ± 14.8. Accordingly, the minimum sample size at a 5% level of significance and 95% power of the study was 22 per group (we included 25 per group for convenience). The total minimum sample size required was 50. The sample size formula used is as follows:

$n = \frac{2 (Z_{\alpha/2} + Z_{\beta})^2 \sigma^2}{d^2}$

where n = sample size required per group, Z_α/2_ = standard normal deviate corresponding to a 5% level of significance, Z_β_ = standard normal deviate corresponding to 95% power, σ = pooled standard deviation, and d = expected mean difference between the two groups.

Rehabilitation protocol

Stroke survivors were randomly assigned to one of the two groups using computer-generated random numbers. A randomization sequence was generated using the Microsoft Excel random function (Microsoft Corp., Redmond, WA, USA). Each eligible participant was assigned a unique identification number. The computer program generated a random sequence that assigned participants to either group in a 1:1 ratio.

Group A was given dual-task training, and Group B was given single-task training. Both groups received a standard conventional exercise program as well.

Group A (Dual-Task Training Group)

Stroke survivors underwent dual-task training, performing motor and cognitive tasks simultaneously. Training activities included motor + motor tasks (15 minutes), such as walking while holding a tray, standing while holding a ball in one hand, and stepping up and down from a 15 cm platform with alternating feet while waving a rattle. Motor + cognitive tasks (15 minutes) included walking while counting numbers forward, standing while reciting days of the week or year in reverse order, and stepping up and down from a 15 cm platform with alternating feet while reciting three digits in reverse order (e.g., 1-2-3:3-2-1).

Group B (Single-Task Training Group)

Stroke survivors in this group engaged in single-task training, focusing solely on motor skills such as walking or standing, without concurrent cognitive tasks. Training activities (30 minutes) included walking, standing and balancing, and stepping up and down from a 15 cm platform with alternating feet.

Both groups participated in their respective training protocols three times per week, with each session lasting 30 minutes, over a total of four weeks. Sessions were supervised in person when feasible, including once-weekly institution-based supervision in the presence of a physiatrist. On other days, participants were monitored remotely via video conferencing or telephone calls to ensure adherence to the prescribed exercise protocol.

Outcome measures

Outcome measures included improvements in motor function, gait, and balance, assessed by:

Timed Up and Go Test (TUG)

The TUG measures functional mobility and dynamic balance. Participants were instructed to stand up from a standard chair, walk three meters, turn, return, and sit down. Time in seconds was recorded. Lower time indicates better performance.

10-Meter Walk Test (10MWT)

The 10MWT assesses gait speed. Participants walked a measured 10-meter walkway at a comfortable speed. Time taken was recorded and gait speed calculated. Lower time indicates better walking performance.

Figure of 8 Walk Test (F8WT)

The F8WT evaluates walking coordination and curved path mobility. Participants walked in a figure-of-8 pattern around two markers placed at a fixed distance. Completion time in seconds was recorded. Lower time indicates better coordination and balance.

Statistical analysis

Data were analyzed using the IBM SPSS Statistics for Windows, Version 25 (Released 2017; IBM Corp., Armonk, New York, United States). Normality of distribution for continuous variables was assessed using the Shapiro-Wilk test. As the outcome variables were not normally distributed, continuous variables are presented as median and interquartile range (IQR). Categorical variables are presented as numbers and percentages.

Between-group comparisons were performed using the Mann-Whitney U test. Within-group comparisons across time points were analyzed using the Wilcoxon signed-rank test. Categorical variables were compared using the chi-square test or Fisher's exact test. A p-value of less than 0.05 was considered statistically significant.

## Results

Sixty stroke survivors were enrolled, with 30 participants in each group; all randomized participants completed the intervention and follow-up. Comprehensive demographic details of the study participants are outlined in Table 1. The mean age in group A was 50 ± 10 years, while that in group B was 50 ± 11 years, with a predominant male representation in both groups (87%, 80%). Most participants had an ischemic type of stroke in both groups (70% in group A and 63% in group B). Both groups were comparable in terms of age, gender distribution, and stroke type.

**Table 1:** Participant demographics Values are presented as mean ± standard deviation or number (percentage). An independent sample t-test was used for continuous variables, and a chi-square test was used for categorical variables.

Characteristic	Group A (dual task)	Group B (single task)	Test statistics	p-value
Number of participants	30	30	-	-
Age (years, mean ± SD)	50 ± 10	50 ± 11	t = 0.37	0.71
Gender (male), n (%)	26 (87)	24 (80)	χ^2^ = 0.12	0.73
Type of stroke, n (%)				
Ischemic	21 (70)	19 (63)	χ^2^ = 0.30	0.58
Hemorrhagic	9 (30)	11 (37)	χ^2^ = 0.30	0.58

Outcomes

Both groups showed improvements throughout the study; however, the dual-task training group exhibited more substantial improvements in motor function tests. The summary of outcome measures at baseline and post-intervention is delineated in Table 2.

**Table 2:** Summary of outcome measures at baseline and postintervention Values are presented as median (interquartile range (IQR)). Between-group comparisons were performed using the Mann-Whitney U test.

Outcome measure	Group A (dual task)	Group B (single task)	Statistical test	Test statistic (U)	p-value
Timed Up and Go Test (seconds)					
Baseline	14.08 (10.92-17.54)	13.68 (11.168-15.57)	Mann-Whitney U	398	0.442
4 weeks	9.97 (9.03-12.308)	12.79 (10.208-14.622)	Mann-Whitney U	263	0.035
8 weeks	9.03 (8.308-10.948)	11.28 (8.41-13.16)	Mann-Whitney U	286.5	0.084
10-Meter Walk Test (seconds)					
Baseline	12.21 (10.508-15.702)	11.34 (9.80-13.85)	Mann-Whitney U	367.5	0.223
4 weeks	10 (8.905-11.065)	11.6 (9.055-12.792)	Mann-Whitney U	282.5	0.073
8 weeks	8.61 (7.602-9.54)	10.26 (8.518-11.145)	Mann-Whitney U	251.5	0.021
Figure of 8 Walk Test (seconds)					
Baseline	12.31 (10.11-17.26)	13.04 (9.368-13.965)	Mann-Whitney U	417.5	0.631
4 weeks	10.6 (9.008-11.61)	12.41 (9.305-13.43)	Mann-Whitney U	268	0.042
8 weeks	9.9 (7.99-11.15)	11.52 (8.525-13.003)	Mann-Whitney U	266	0.039

Within-group comparisons across time points are presented in Table 3. Using the Wilcoxon signed-ranked test, both the dual-task and single-task groups demonstrated statistically significant improvements in the TUG, 10MWT, and F8WT at both four and eight weeks compared to baseline (all p < 0.001). These findings indicate sustained improvements over time within each group.

**Table 3:** Outcome-specific intragroup comparisons across time points Within-group comparisons were performed using the Wilcoxon signed-rank test.

Outcome	Group	Comparison	Statistical test	Z value	p-value
Timed Up and Go Test (TUG)	Dual task	Baseline vs. 4 weeks	Wilcoxon signed-rank	-4.623	<0.001
		Baseline vs. 8 weeks	Wilcoxon signed-rank	-4.623	<0.001
	Single task	Baseline vs. 4 weeks	Wilcoxon signed-rank	-4.054	<0.001
		Baseline vs. 8 weeks	Wilcoxon signed-rank	-4.623	<0.001
10-Meter Walk Test (10MWT)	Dual task	Baseline vs. 4 weeks	Wilcoxon signed-rank	-4.623	<0.001
		Baseline vs. 8 weeks	Wilcoxon signed-rank	-4.623	<0.001
	Single task	Baseline vs. 4 weeks	Wilcoxon signed-rank	-4.623	<0.001
		Baseline vs. 8 weeks	Wilcoxon signed-rank	-4.623	<0.001
Figure of 8 Walk Test (F8WT)	Dual task	Baseline vs. 4 weeks	Wilcoxon signed-rank	-4.623	<0.001
		Baseline vs. 8 weeks	Wilcoxon signed-rank	-4.623	<0.001
	Single task	Baseline vs. 4 weeks	Wilcoxon signed-rank	-4.623	<0.001
		Baseline vs. 8 weeks	Wilcoxon signed-rank	-4.623	<0.001

## Discussion

The present study investigated the effects of four weeks of dual-task and single-task training, including a cognitive task component, on cognitive function and walking ability in stroke patients, using functional evaluation tools. The subjects were assigned to the dual-task training and single-task training groups, and the difference between pre-training and post-training was compared within and between the two groups.

Our study's results suggest that dual-task training may provide additional improvements in motor function than single-task training in stroke survivors. Participants in the dual-task group showed notable progress in the TUG, 10MWT, and F8WT, suggesting potential benefits of combining cognitive tasks with motor exercises. These significant gains in functional performance suggest that dual-task training not only supports motor recovery but also helps manage cognitive-motor interference, a frequent challenge in stroke rehabilitation.

Our findings indicated that both groups experienced a reduction in TUG completion time by the end of the training period and at follow-up (eight weeks), with the dual-task group showing a trend towards greater improvements than the single-task group. Throughout the training period, TUG time decreased consistently in both groups, with the dual-task group demonstrating a somewhat greater reduction than the single-task group. An et al. observed a similar decline in TUG time across three groups (motor dual-task training, cognitive dual-task training, and motor-cognitive dual-task training) at the conclusion of the training period, with the motor-cognitive dual-task group showing significant differences compared to the other two groups [12]. Another study by Kim et al. used TUG to assess dynamic balance in stroke patients and found significant differences between the pre- and post-intervention (four weeks) periods in the dual-task and single-task training groups. Additionally, there were significant differences between the pre-intervention and follow-up periods (six weeks) [9].

In our study, we compared gait speed between the two groups using the 10MWT. We found that the dual-task group showed somewhat greater improvements in three types of motor dual and three types of motor plus cognitive dual tasks compared to the single-task group at four and eight weeks, respectively. This aligns with the findings of An et al., who reported that the motor and cognitive dual-task gait training group (MCDGT) showed significant improvement in gait speed after performing five types of motor dual tasks and five sets of cognitive dual tasks, each for 15 minutes, while maintaining gait on the treadmill [12].

In the F8WT used in our study, we observed that the dual-task group showed greater improvement in gait speed than the single-task group. This finding is consistent with previous reports by Kim et al., who found that stroke patients showed better weight support and weight shifting in both legs after completing curved walking training in both directions on a figure-of-8 shaped track [9]. Ko et al. also reported that stroke patients' walking ability and speed significantly improved through exercise regimens that enhance proprioception on the affected side [13]. In our study, patients in the dual-task group were asked to walk while holding a tray (motor-motor dual task), compared with simple walking in the single-task group. Typically, the Indian population resides in smaller homes, and these exercise programs could have been conducted in limited spaces, requiring greater maneuverability and greater motor and cognitive input. This may have contributed to the improvements in the F8WT parameters in the dual-task group compared to the single-task group.

This outcome differs from that of the study by Kim et al., which observed no differences between the two groups in improvements over time in F8WT, as assessed by speed and step count. This was likely due to insufficient intervention in the gait training program components involving curved paths in their research [9].

While pooled data show dual-task training improves gait speed and spatiotemporal metrics, effects on global balance scales (e.g., Berg Balance Scale) or composite motor scales are sometimes small or non-significant. Our study showed robust changes in F8WT and 10MWT but a smaller between-group effect for TUG by week 8. This divergence is not unique: TUG is a composite task (sit-to-stand, gait, turn, sit) influenced by multiple domains; improvements in gait speed may not fully translate to faster multicomponent tasks once cognitive load is removed or when turning/transfer demands predominate.

The concept of "cognitive reserve," which suggests that testing the brain's cognitive abilities can enhance its adaptability, might explain the superior results of the dual-task group. Unlike motor training alone, this is likely facilitated by the activation of both motor and cognitive pathways, leading to a more significant reorganization of brain circuits. Neuroimaging studies, such as those by Beurskens and colleagues [14], have shown increased connectivity in the prefrontal cortex and motor areas during dual-task activities, supporting this theory. Our findings align with those of He et al., who discovered that dual-task training improved walking speed and balance in stroke patients more effectively than traditional therapy [15].

Incorporating dual-task training into standard clinical practice could enhance outcomes and reduce rehabilitation times, thereby decreasing the need for long-term care and associated medical costs. Patients may experience greater independence and an improved quality of life due to enhanced motor and cognitive recovery from such training. To optimize recovery trajectories, dual-task training should be introduced early in the rehabilitation process, potentially during the acute or subacute stages following a stroke, as observed in our study.

Limitations and future research directions

While promising, our findings should be interpreted in the context of several limitations. The relatively short duration of the intervention and follow-up period may not fully capture the long-term sustainability of the observed benefits. Additionally, certain factors, such as participants’ daily activities and adherence outside the supervised sessions, could not be fully controlled and may have influenced functional outcomes. The modest sample size also limited the ability to perform subgroup analyses, including stratification based on stroke localization. Furthermore, methodological aspects such as the absence of assessor blinding and limited reporting of allocation concealment procedures may affect the generalizability of the findings.

Further research should be conducted to explore the differential effects of various types of cognitive tasks used in dual-task training, identify the most effective combinations, investigate the effects of extended dual-task training programs, and include larger, more heterogeneous cohorts to validate and expand upon these findings. Long-term follow-up studies are essential to assess the persistence of improvements and to evaluate the optimal duration and intensity of dual-task interventions.

## Conclusions

In conclusion, both dual-task and single-task training resulted in improvements in gait speed, dynamic balance, and functional mobility among stroke survivors. Though dual-task training showed relatively greater improvement compared to single-task training, not all between-group differences were significant at the eight-week follow-up. These findings suggest that incorporating cognitive elements into motor rehabilitation may provide additional benefits for improving functional performance after stroke. Further studies with larger samples and longer follow-up durations are needed to confirm the long-term effectiveness of dual-task training in stroke rehabilitation.
